# Pre-infarction Angina: Time Interval to Onset of Myocardial Infarction and Comorbidity Predictors

**DOI:** 10.3389/fcvm.2022.867723

**Published:** 2022-05-26

**Authors:** Yohei Sotomi, Yasunori Ueda, Shungo Hikoso, Katsuki Okada, Tomoharu Dohi, Hirota Kida, Bolrathanak Oeun, Akihiro Sunaga, Taiki Sato, Tetsuhisa Kitamura, Hiroya Mizuno, Daisaku Nakatani, Yasuhiko Sakata, Hiroshi Sato, Masatsugu Hori, Issei Komuro, Yasushi Sakata

**Affiliations:** ^1^Department of Cardiovascular Medicine, Osaka University Graduate School of Medicine, Osaka, Japan; ^2^Cardiovascular Division, National Hospital Organization Osaka National Hospital, Osaka, Japan; ^3^Department of Transformative System for Medical Information, Osaka University Graduate School of Medicine, Osaka, Japan; ^4^Division of Environmental Medicine and Population Sciences, Department of Social and Environmental Medicine, Graduate School of Medicine, Osaka University, Osaka, Japan; ^5^Department of Clinical Medicine and Development and Department of Cardiovascular Medicine, National Cerebral and Cardiovascular Center, Suita, Japan; ^6^School of Human Welfare Studies Health Care Center and Clinic, Kwansei Gakuin University, Hyogo, Japan; ^7^Osaka International Cancer Institute, Osaka, Japan; ^8^Department of Cardiovascular Medicine, University of Tokyo Graduate School of Medicine, Tokyo, Japan

**Keywords:** acute myocardial infarction, pre-infarction angina, prevention, public education, real-world

## Abstract

**Aims:**

As part of efforts to identify candidates for patient education aimed at decreasing mortality from acute myocardial infarction, we investigated the prevalence of pre-infarction angina and its predictors among comorbidities in patients who were hospitalized with acute myocardial infarction (MI).

**Methods:**

We conducted a prospective multicenter observational registry of MI patients from 1998 to 2014 (*N* = 12,093). The present study investigated the prevalence of pre-infarction angina and its predictors among comorbidities with a logistic regression model. Pre-infarction angina was defined as chest pain/oppression observed within 1 month before the onset of MI but which lasted <30 min.

**Results:**

After excluding 976 (8.1%) patients with missing data on pre-infarction angina, 11,117 patients [66.4 ± 12.0 years, 9,096 (75.2%) male] were analyzed. Of these, 5,428 patients (48.8%) experienced pre-infarction angina before the onset of MI, while 5,689 (51.2%) experienced sudden onset of acute MI. Most patients experienced the first episode of angina >6 h before the onset of MI, while 15% did so ≤6 h before. Patients with hypertension, diabetes, dyslipidemia, or a family history of MI had a higher probability of pre-infarction angina than those without. Elderly patients and those with a history of cerebrovascular disease were less likely to experience pre-infarction angina.

**Conclusions:**

Almost half of MI patients in our registry experienced pre-infarction angina before MI onset. Patients with hypertension, diabetes, dyslipidemia, or a family history of MI had a higher probability of experiencing pre-infarction angina than those without.

## Introduction

The in-hospital mortality rate of patients with acute myocardial infarction (MI) has improved to <10% due to advanced treatment after hospital admission, including primary percutaneous coronary intervention (1–3). Nevertheless, many cardiac arrests and deaths occur out-of-hospital, and acute MI remains a life-threatening condition worldwide (4). Once a person suffers acute MI, the overall mortality rate is 30%−40%, and the out-of-hospital mortality rate is 25%−30% (5). Indeed, as many as 77% of overall deaths from coronary heart disease occur out of hospital (1). Some MI patients who die before reaching a hospital likely experience prodrome before the onset of MI. Appropriate medical contact in this pre-infarction angina phase may prevent the onset of MI and save lives (5). However, this requires that patients be able to recognize their symptoms as pre-infarction angina, which in turn highlights the importance of public education in encouraging timely medical consultation. Nevertheless, which MI patients are more likely to experience pre-infarction angina remains unclear.

Many public campaigns to prevent disease are underway, including “Know Diabetes by Heart” by the American Heart Association and American Diabetes Association and the “STOP MI Campaign” by the Japanese Circulation Society. The scope and reach of these campaigns will be expanded by the upcoming internet of things (IoT) technology (6, 7). Examples include the possibility of detecting ischemia using smart devices (8). For MI, such campaigns will best be targeted at populations with a higher probability of pre-infarction angina. However, it is still unknown which comorbidities are likely to accompany pre-infarction angina in these patients.

Here, to identify suitable candidates for public education, we investigated the prevalence of pre-infarction angina and its predictors among comorbidities.

## Methods

### Study Population

We used the Osaka Acute Coronary Insufficiency Study (OACIS) database (*N* = 12,093) to investigate (1) the prevalence of pre-infarction angina, (2) time to onset of MI, and (3) associations between patients' comorbidities and pre-infarction angina. The OACIS was a prospective, multicenter observational study designed to collect and analyze demographic, procedural, and outcome data in patients with acute MI at 25 collaborating hospitals with cardiac emergency units. Patients were enrolled from 1998 to 2014 and followed until 2019. Written informed consent was obtained from each patient (9, 10). A diagnosis of acute MI was made if the patient fulfilled at least two of the following three criteria: (1) history of central chest pressure, pain, or tightness lasting ≥30 min, (2) typical ECG changes (ie, ST-segment elevation ≥0.1 mV in one standard limb lead or two precordial leads, ST-segment depression ≥0.1 mV in two leads, abnormal Q waves, or T-wave inversion in two leads), and (3) a rise in serum creatinine phosphokinase concentration to more than twice the normal laboratory value (11, 12). All 25 collaborating hospitals were encouraged to enroll consecutive patients with acute MI.

For the present study, we prospectively collected OACIS data obtained by research cardiologists and trained research nurses using a specific reporting form. The OACIS study (and any subanalyses) are registered with the UMIN-CTR (University Hospital Medical Information Network Clinical Trials Registry) in Japan (ID: UMIN000004575). The study protocol complied with the Helsinki Declaration. The study was approved by the ethics committee of Osaka University Hospital (approval number: 14360).

### Definition of Pre-infarction Angina and Onset of MI

Pre-infarction angina was defined as chest pain/oppression within 1 month before the onset of MI which lasted <30 min. Only typical chest pain was considered as pre-infarction angina; atypical symptoms such as shortness of breath, diaphoresis, fatigue, or pain at a site other than the chest were neither considered as pre-infarction angina nor recorded in this study. The presence and characteristics of typical chest pain were actively enquired about during registration. Onset of MI was defined as the start of chest pain which lasted ≥30 min. Time interval from the first episode of pre-infarction angina to the onset of MI was prospectively collected.

### Statistical Analysis

Eligible patients were stratified by the presence or absence of pre-infarction angina. Categorical variables are expressed as counts (percentages) and compared with the Chi-squared test or Fisher exact test. Continuous variables are expressed as median (interquartile range) and compared using the Mann–Whitney *U*-test. Data are presented by listwise deletion. Serial change in the prevalence of pre-infarction angina from 1998 to 2014 was evaluated with the Cochran-Armitage trend test. We used a logistic regression model to investigate the relationship between patient comorbidities and pre-infarction angina. The following factors were selected based on clinician consensus and included in the model: age, sex, hypertension, smoking, prior MI, family history of MI, cerebrovascular disease, cancer, arteriosclerosis obliterans (ASO), hemoglobin per 1 g/dl, creatinine per 0.1 mg/dl, low density lipoprotein cholesterol (LDL-C) per 10 mg/dl, and HbA1c per 1.0%. Because the exclusion of cases with missing data could have caused bias in this analysis and loss of power in detecting statistical differences, missing data were imputed by random forest imputation using the “missForest” package prior to the logistic regression analysis. All statistical analyses were performed with R software (version 4.0.5; R Foundation for Statistical Computing, Vienna, Austria).

## Results

### Study Subjects

**Figure 1:**
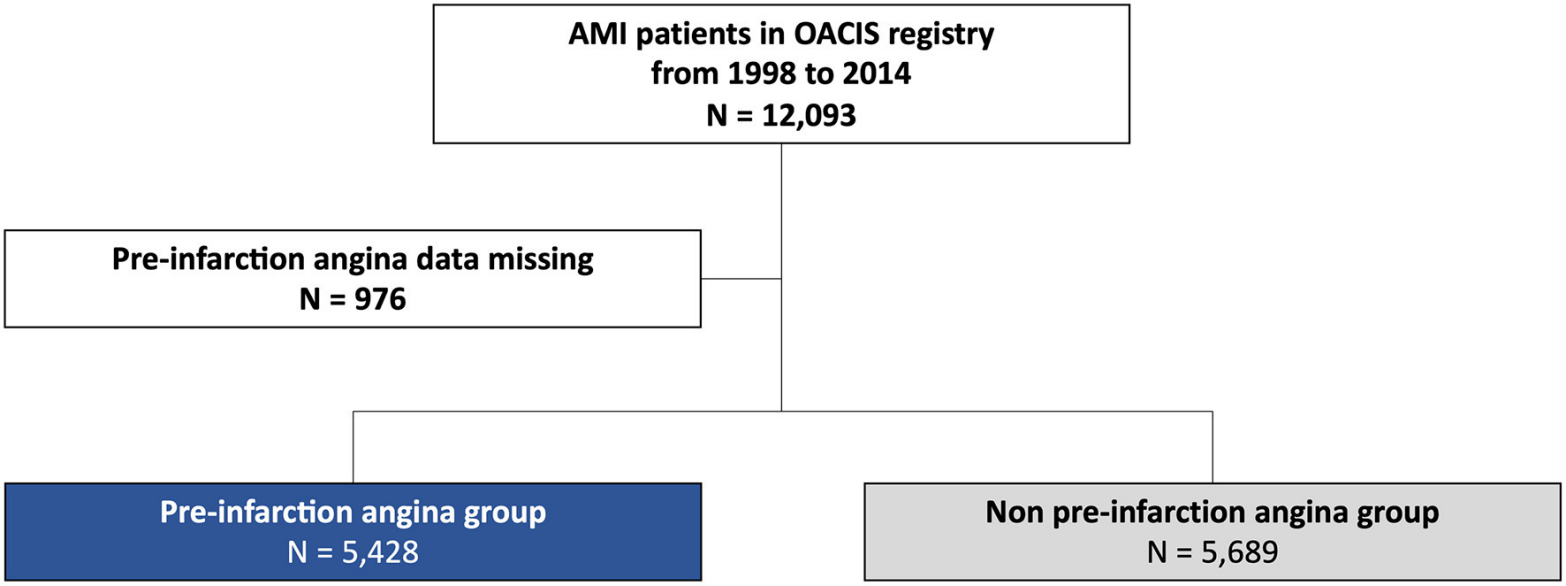
Study flowchart.

After excluding 976 patients (8.1%) with missing data on pre-infarction angina, 11,117 patients [66.4 ± 12.0 years, 9,096 (75.2%) male] were analyzed (Figure 1). Baseline characteristics of patients excluded vs. included in the present analysis are tabulated in Supplemental Table 1. Patients excluded from the analysis were older, less likely to be male, and showed a higher prevalence of diabetes mellitus, hypertension, and chronic kidney disease and a lower prevalence of dyslipidemia and smoking than those analyzed. Of the 11,117 patients analyzed, 5,428 patients (48.8%) experienced pre-infarction angina before the onset of MI, while 5,689 patients (51.2%) experienced sudden onset of acute MI. Baseline demographics of patients with and without pre-infarction angina are tabulated in Table 1. Patients with pre-infarction angina showed a higher prevalence of dyslipidemia, more frequently had a family history of MI, and less frequently had a history of cerebrovascular disease than those without. Peak CK level was significantly lower in patients with pre-infarction angina than in those without (13).

**Table 1:** Baseline characteristics.

	**Pre-infarction angina (+)**	**Pre-infarction angina (–)**	***P*-Value**	**Missing (%)**
Patients, *n*	5,428	5,689		
Age, years	66.00 [57.00, 74.00]	67.00 [59.00, 75.00]	<0.001	0
Male sex	4,126 (76.0)	4,316 (75.9)	0.886	0
Body mass index	23.53 [21.51, 25.78]	23.42 [21.30, 25.65]	0.001	6.4
Diabetes mellitus	1,843 (34.7)	1,812 (32.9)	0.043	3.3
Hypertension	3,242 (61.5)	3,315 (60.2)	0.166	3.7
Dyslipidemia	2,393 (46.0)	2,309 (42.5)	<0.001	5.1
Smoking	3,415 (64.1)	3,510 (63.7)	0.648	3.3
Chronic kidney disease	384 (7.3)	404 (7.4)	0.921	3.6
Prior myocardial infarction	613 (11.6)	652 (11.7)	0.902	2.8
Family history of MI	526 (14.0)	441 (11.3)	0.001	31.1
Cerebrovascular disease	418 (7.9)	569 (10.4)	<0.001	3.6
Cancer	276 (5.2)	346 (6.3)	0.020	3.6
ASO	127 (2.4)	147 (2.7)	0.415	3.6
Hemoglobin, g/dl	14.10 [12.50, 15.30]	13.80 [12.30, 15.10]	<0.001	52.0
Creatinine, mg/dl	0.83 [0.70, 1.06]	0.90 [0.70, 1.11]	<0.001	30.4
Low density lipoprotein cholesterol, mg/dl	124.00 [100.75, 148.10]	118.00 [94.00, 142.25]	<0.001	63.9
HbA1c, %	5.60 [5.20, 6.60]	5.50 [5.10, 6.40]	<0.001	27.2
ST-elevation myocardial infarction	4,450 (83.5)	4,847 (86.7)	<0.001	1.8
Culprit vessel				
Right coronary artery	1,633 (32.4)	1,987 (38.7)	<0.001	8.9
Left anterior descending artery	2,564 (50.8)	2,299 (44.8)	<0.001	8.9
Left circumflex artery	843 (16.7)	765 (14.9)	0.015	8.9
Left main trunk	135 (2.7)	132 (2.6)	0.798	8.9
Peak CK, IU/L	1,801.00 [828.00, 3,512.50]	2,093.00 [994.25, 4,024.00]	<0.001	6.0
Peak CK-MB, IU/L	163.00 [72.00, 315.00]	181.00 [85.17, 356.00]	<0.001	14.0

*Data are expressed as median [interquartile range] or number (percentage)*.

*ASO, arteriosclerosis obliterans; CK, creatine kinase; CK-MB, creatine kinase myocardial band; MI, myocardial infarction*.

### Prevalence of Pre-infarction Angina

**Figure 2:**
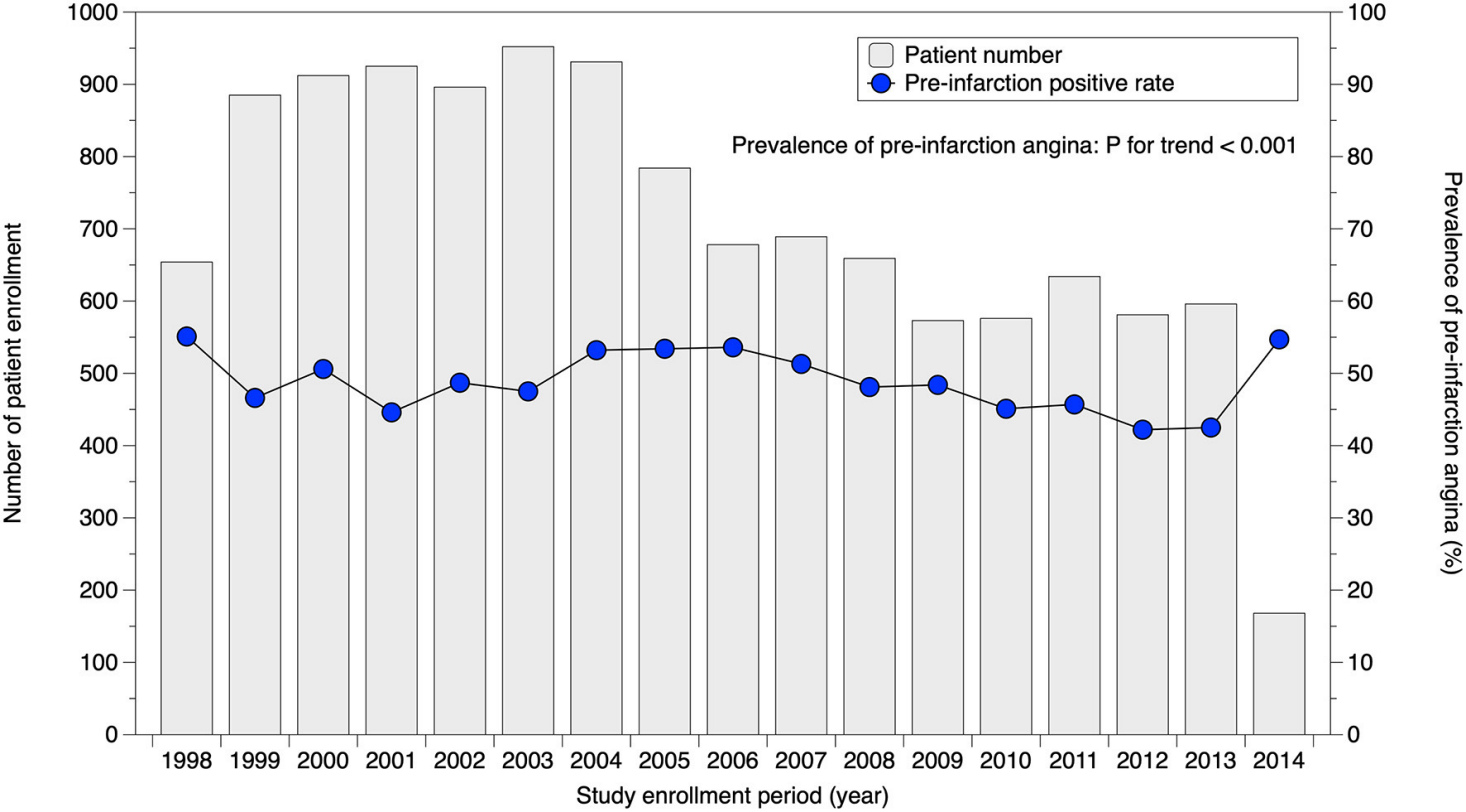
Serial change in prevalence of pre-infarction angina. Prevalence of pre-infarction angina slightly but significantly decreased over time (*P* for trend <0.001).

Serial change in the prevalence of pre-infarction angina is illustrated in Figure 2. During the study period from 1998 to 2014, prevalence gradually decreased (*P* for trend <0.001). Crude rates of pre-infarction angina in specific patient subgroups stratified by sex are tabulated in Table 2. The prevalence in male patients in their 50s, for example, was 52.2%. In both sexes, patients in their 80s had a lower prevalence of pre-infarction angina than younger patients. Patients with dyslipidemia showed a higher prevalence of pre-infarction angina than those without in both sexes, whereas patients with a history of cerebrovascular disease showed a lower prevalence than those without.

**Table 2:** Crude rates of pre-infarction angina.

	**Male**	**Female**
	***N* = 8,442**	***N* = 2,674**
Overall	4,126/8,442 (48.9)	1,302/2,674 (48.7)
Age		
20s−40s	473/921 (51.4)	40/82 (48.8)
50s	1,038/1,989 (52.2)	114/210 (54.3)
60s	1,326/2,744 (48.3)	372/687 (54.1)
70s	993/2,073 (47.9)	470/925 (50.8)
80s-	295/713 (41.4)	306/770 (39.7)
*P*-value^*^	<0.001	<0.001
Hypertension (**–**)	1,630/3,404 (47.9)	400/820 (48.8)
Hypertension (+)	2,372/4,782 (49.6)	870/1,774 (49.0)
*P*-value^†^	0.131	0.935
Diabetes mellitus (**–**)	2,653/5,461 (48.6)	810/1,701 (47.6)
Diabetes mellitus (+)	1,378/2,756 (50.0)	465/899 (51.7)
*P*-value^†^	0.233	0.051
Dyslipidemia (**–**)	2,178/4,577 (47.6)	629/1,360 (46.2)
Dyslipidemia (+)	1,778/3,509 (50.7)	615/1,192 (51.6)
*P*-value^†^	0.006	0.008
Smoking (**–**)	996/2,039 (48.8)	915/1,874 (48.8)
Smoking (+)	3,055/6,209 (49.2)	360/715 (50.3)
*P*-Value^†^	0.800	0.516
Family history of MI (**–**)	2,493/5,152 (48.4)	750/1,542 (48.6)
Family history of MI (+)	412/755 (54.6)	114/212 (53.8)
*P*-Value^†^	0.002	0.184
Cerebrovascular disease (**–**)	3,688/7,466 (49.4)	1,163/2,307 (50.4)
Cerebrovascular disease (+)	314/706 (44.5)	104/281 (37.0)
*P*-Value^†^	0.014	<0.001

*Data are presented as number (percentage)*.

* *P value indicates comparison in different ages. Multiple pairwise comparison with the Bonferroni correction (P-values < 0.005 were considered statistically significant) showed that (1) in male patients, those in their 80s had a significantly lower prevalence of pre-infarction angina than those in their 20–40s and 50s; (2) in female patients, those in their 80s had a lower prevalence of pre-infarction angina than those in their 50s, 60s, and 70s. The other comparisons were not statistically significant*.

† *P value indicates comparison between patients with and without the comorbidity*.

*MI, myocardial infarction*.

**Figure 3:**
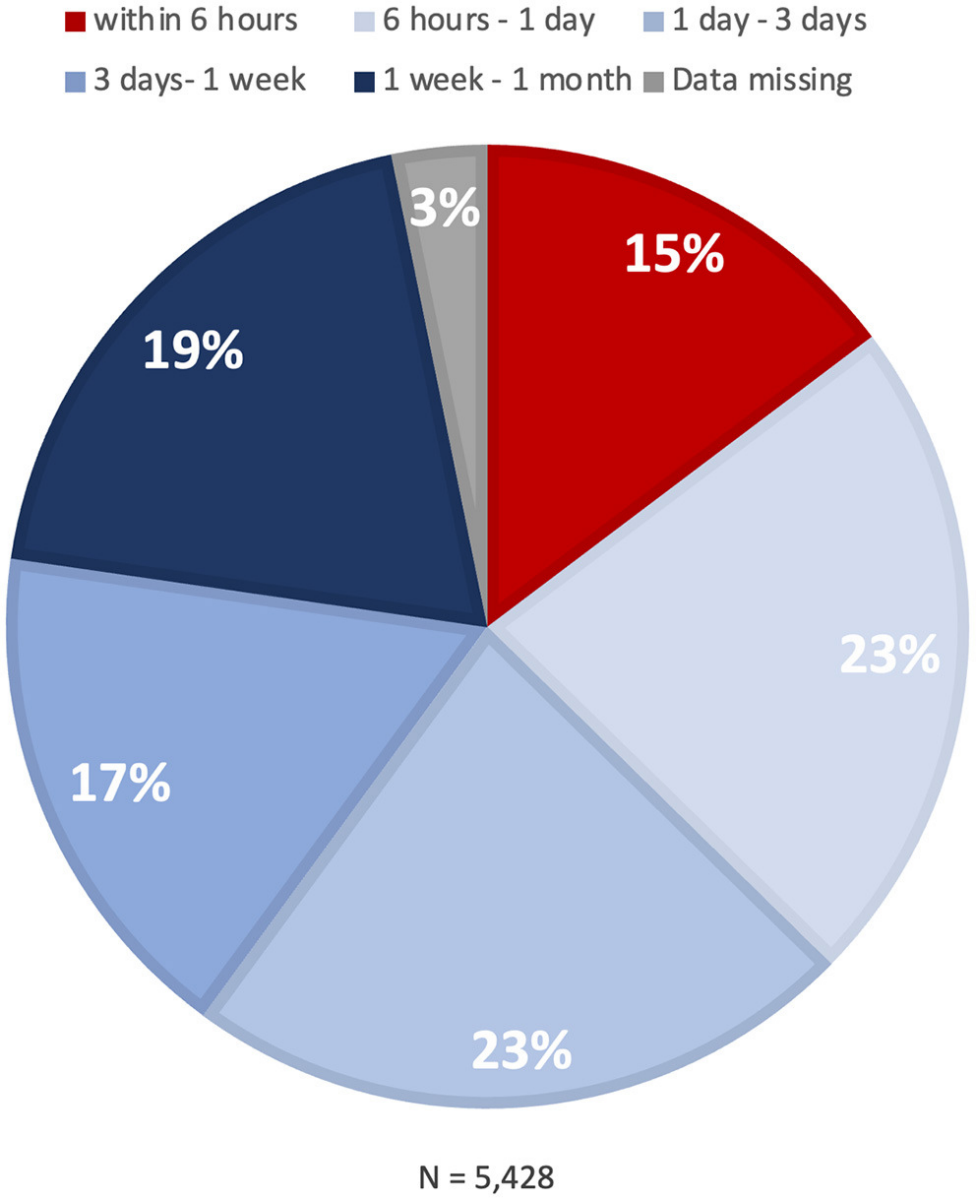
Time to onset of myocardial infarction. A pie chart indicates the distribution of time from the onset of pre-infarction angina to the onset of myocardial infarction.

Time from the first episode of pre-infarction angina to the onset of MI is summarized in Figure 3. Time to MI onset was >6 h for most patients, vs. ≤6 h for 15%.

### Predictors of Pre-infarction Angina

**Figure 4:**
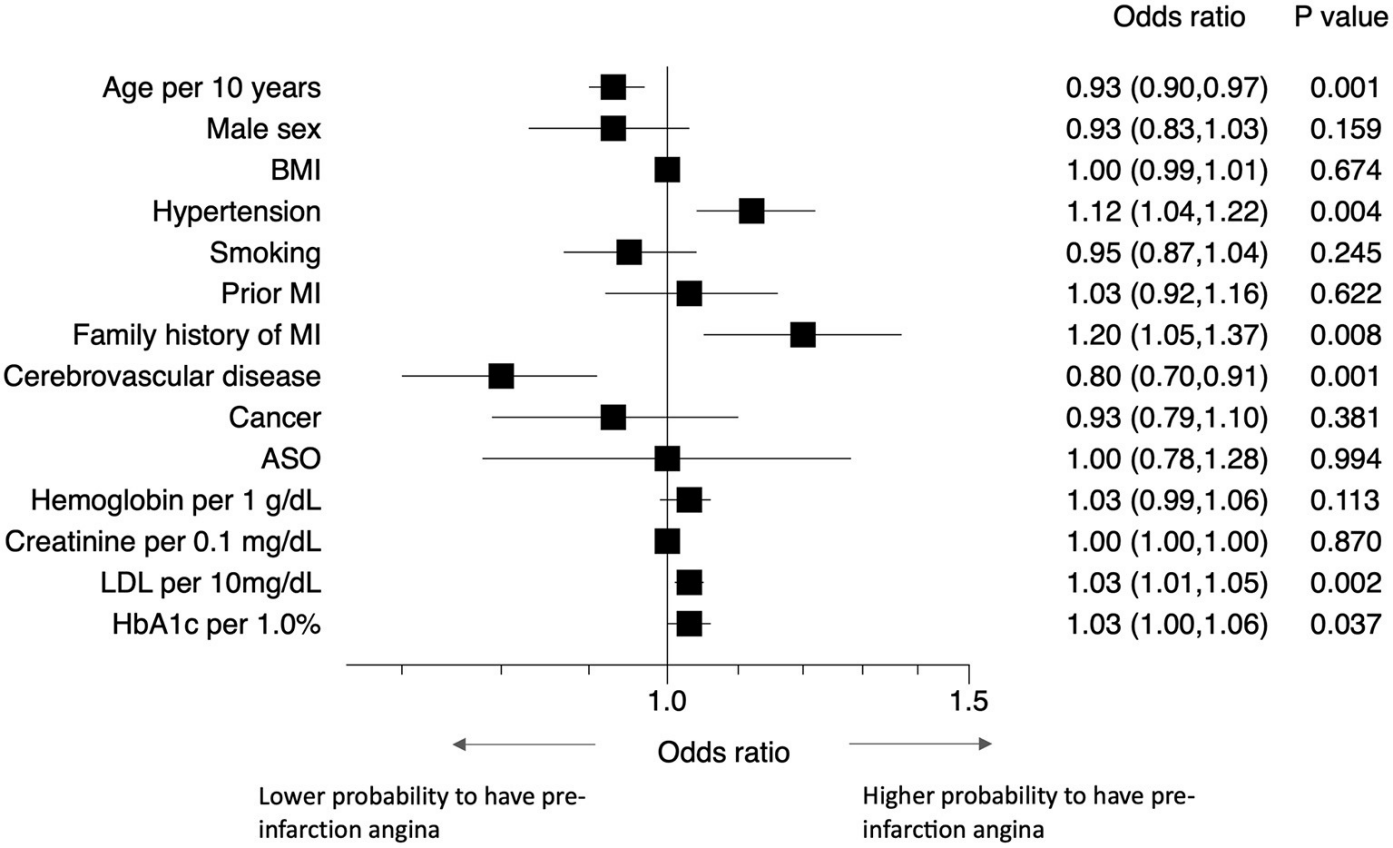
Predictors of pre-infarction angina. Forest plots showing the odds ratios of having pre-infarction angina with various comorbidities. ASO, arteriosclerosis obliterans; BMI, body mass index; LDL-C, low density lipoprotein cholesterol; MI, myocardial infarction.

Patients with hypertension, diabetes, dyslipidemia, or a family history of MI had a higher probability of experiencing pre-infarction angina than those without (Figure 4). Elderly patients and those with a history of cerebrovascular disease were less likely to experience pre-infarction angina.

## Discussion

In this study, we used data from a large-scale prospective observational registry to investigate the prevalence of pre-infarction angina and its related comorbidities. Approximately half of the MI patients in the registry experienced pre-infarction angina before the onset of MI. In most patients, the time from the onset of angina to that of MI was >6 h, but was ≤6 h in 15%. Patients with hypertension, diabetes, dyslipidemia, or a family history of MI had a higher probability of pre-infarction angina than those without. Further, elderly patients and those with a history of cerebrovascular disease were less likely to experience pre-infarction angina.

### Prevalence of Pre-infarction Angina and Time Interval to the Onset of MI

Although a number of studies have reported the prevalence of pre-infarction angina, most had relatively small cohorts and a retrospective design (4, 14–18). Results varied from approximately 30%−55%, presumably because of different definitions of pre-infarction angina. Our present analysis provides the first prospective evidence for pre-infarction angina rates, as collected in the largest cohort with a pre-defined definition under current practice standards. Approximately half of the MI patients experienced pre-infarction angina within 1 month before the onset of MI. Of note, this study investigated the prevalence of pre-infarction angina in patients who were admitted to hospital for acute MI. This is not strictly the same as the true prevalence of pre-infarction angina for all MI patients. As presented in Figure 2, prevalence in our cohort over time gradually but significantly decreased (*P* for trend <0.001). This implies that recent patients with pre-infarction angina were treated at the unstable angina stage more frequently than before, assuming that the true prevalence of pre-infarction angina did not change over time. Nevertheless, the decrease was markedly limited, and improvement with public education appears likely.

Although time interval from pre-infarction angina to MI onset has been reported, the study was conducted about five decades ago (18). Among 99 patients who experienced chest pain before MI, six patients (6%) experienced warning symptoms within 24 h of major infarction, while the majority (94%) did so 1 day or more before. Our study (*N* = 11,117) showed that approximately 60% of patients with pre-infarction angina have sufficient time to see a doctor before MI onset (>24 h). For the remaining 40%, in contrast, MI occurred within 24 h after the onset of pre-infarction angina. Of note, 15 percent of the MI patients had ≤6 h from pre-infarction angina to onset, which would favor the earliest possible medical contact. It is likely that the different proportions between the present and previous finding are attributable to different definitions of pre-infarction angina.

### Possible Mechanisms of Pre-infarction Angina and Its Predictors

The pathology of MI is likely related to the presence or absence of pre-infarction angina, although in the present series we do not have data on the pathology of MI in individual cases. The presence of pre-infarction angina may imply that the coronary artery was gradually occluded, whereas its absence might suggest that the coronary artery was suddenly occluded. In patients with pre-infarction angina, MI is likely to develop from severe coronary stenosis *via* unstable angina or mild stenosis due to rupture of vulnerable plaque with gradual thrombus formation. A pathological study indicated that coronary occlusion is often preceded by a variable period of plaque instability and thrombus formation, initiated days or weeks before total occlusion (19). In patients without pre-infarction angina, MI is likely to develop from mild stenosis with vulnerable plaque and its rupture or erosion and subsequent sudden thrombus formation. A pathology study showed that fresh thrombus (<1 day) was present in approximately half (49%) of MI patients (19). This is closely similar to the proportion with pre-infarction angina in the current study. Gradual occlusion may cause “pre-conditioning” for ischemia, which may in turn explain the lower peak cardiac enzyme levels in patients with pre-infarction angina than in those without.

Patients with a family history of MI, hypertension, dyslipidemia, or diabetes had a higher probability of pre-infarction angina than those without. A family history of MI probably indicates that the patients become well-informed on the disease and its symptoms. Hypertension and dyslipidemia are both common risk factors of atherosclerosis. We speculate that these factors inhibit sudden massive thrombus formation. However, the precise mechanism is still unknown and remains to be investigated in future studies. Our finding for diabetes is particularly notable, because it appears to contradict previous findings: these indicated that patients with diabetes mellitus may not report classic ischemic chest pain but may instead present with dyspnea, nausea, fatigue, cough, or other non-specific symptoms due to diabetes-associated cardiovascular autonomic neuropathy (20–22). In contrast, our study showed that diabetes mellitus was a predictor of pre-infarction angina. When we divided patients into those with and without diabetes, diabetic patients more frequently experienced pre-infarction angina (typical chest pain) than non-diabetic patients [50.4 vs. 48.3%, *P* = 0.043]. A number of reasons for this difference can be considered, including differences in race, medical system, and patient education level, as well as the up-to-datedness of treatment for diabetes and lower prevalence of diabetic neuropathy (23) than previously thought (20, 21). Selection bias might also play a role—our assessment was limited to MI patients admitted to hospital, and did not include patients who died out of hospital. This finding should be confirmed in a large-scale study.

We also found that elderly patients and those with a history of cerebrovascular disease were less likely to experience pre-infarction angina. Both of these risk factors may be associated with sensory and motor disorders—this may in turn lead to an increased anginal threshold and lower burden of daily activity, and consequently to the absence of typical angina.

### Clinical Implications and Future Perspectives

Hypertension, diabetes, dyslipidemia, and a family history of MI are all well-known risk factors of coronary artery disease. Nevertheless, our study did show the additional clinical importance of these comorbidities. Patients with them had a higher probability of pre-infarction angina, which can act as a warning of MI before its onset. This point is the novelty of the present findings. Accordingly, patients should be educated to easily recognize pre-infarction angina themselves, as a warning of MI. Atypical symptoms might be difficult to interpret for patients and even physicians. In our study, half of the MI patients experienced “typical” anginal symptoms, which are easy to interpret and therefore a useful aid to prompt diagnosis. Physicians should never dismiss the chance of prompt diagnosis of angina and prevention of MI (18). Rapid action by physicians and patients in the pre-infarction angina phase can prevent the onset of MI in at least half of MI patients. Public education efforts such as the Japanese Circulation Society's “STOP MI Campaign” are helpful, and indeed likely even more efficient in reducing the mortality of MI than in-hospital treatment, given that approximately half of out-of-hospital deaths might be avoided (5). Mortality of MI in Japan decreased after the launch of the STOP MI Campaign in 2014 from 31.1% (2014) to 24.8% (2020), albeit that the reason for this decrease is multifactorial (24, 25).

Social media and smart devices may have major potential in this field (6–8). A public campaign through social media was shown to be effective in reducing COVID-19 infection in the United States (7). In the field of ischemic heart disease, public education through a short message service significantly shortened the onset-to-door time of MI patients (6). As for smart devices, the Apple watch can detect atrial fibrillation and provide patients with an alert (26, 27). We have also noted the increased use of this technology in our clinical practice, with more and more patients presenting at our outpatient clinic with an Apple watch showing an electrocardiogram of atrial fibrillation. In ischemic heart disease also, the possibility of detecting ischemia was recently reported (8). Although the clinical use of this technology in ischemic heart disease has yet to be validated, we consider that it is only a matter of time before it is incorporated into daily clinical practice. Automatic detection of pre-infarction angina and an alert by a smart device may prevent the onset of MI. Public education in combination with advanced smart technology and recent IoT infrastructure must surely be effective. Our present study suggests that patient subpopulations with hypertension, diabetes, dyslipidemia, or a family history of MI are suitable candidates for public education. Such a targeted public campaign in combination with smart devices and social media might show a synergistic effect. This research finding should be implemented in designing future prospective large-scale studies with the upcoming IoT technology.

### Study Limitations

Several limitations of our study should be acknowledged. First, angina as a symptom is subjective, although the definition was pre-defined and the data were prospectively collected. Second, the current population was limited to patients who were admitted to a hospital due to myocardial infarction, and the present findings might not therefore be directly applicable to patients who were not admitted to hospitals, namely out-of-hospital deaths. Third, patients excluded from the present analysis [*N* = 976 (8.1%)] showed significantly different baseline characteristics to those who were analyzed, resulting in a degree of selection bias. Lastly, the generalizability of the findings to other regions and ethnicities is limited by racial differences, differing healthcare systems, etc. in Japan compared with other countries.

In conclusion, we found that half of the MI patients in our registry experienced pre-infarction angina before the onset of MI. Most patients with pre-infarction angina had sufficient time (>6 h) to go to the hospital before MI onset. However, 15% had only ≤6 h, which would favor early medical contact. Patients with hypertension, diabetes, dyslipidemia, or a family history of MI had a higher probability of having pre-infarction angina than those without. This study suggested the need to reappraise these common coronary risk factors. Prompt medical consultation may enable treatment at the pre-infarction angina stage, and thereby be preventive for MI. This patient sub-population may represent a good target for public education. Future prospective large-scale studies with upcoming IoT technology are warranted.

## Data Availability Statement

Our study data will not be made available to other researchers for purposes of reproducing the results because of Institutional Review Board restrictions.

## Ethics Statement

The studies involving human participants were reviewed and approved by Osaka University Hospital. The patients/participants provided their written informed consent to participate in this study.

## Author Contributions

YSo, YU, and SH: concept and design, data analysis and statistical analysis, and manuscript draft. All authors: critical revision, editing, and approval of the final manuscript. All authors contributed to the article and approved the submitted version.

## Funding

This work was supported by Grants-in-Aid for University and Society Collaboration (#19590816, #19390215, and #25461055) from the Japanese Ministry of Education, Culture, Sports, Science and Technology, Tokyo, Japan.

## Conflict of Interest

YSo received research grants from Abbott Medical Japan, and speaker honoraria from Abbott Medical Japan, Boston Scientific Japan, TERUMO, Japan Lifeline, Biosensors, and Medtronic, and is an endowed chair funded by TOA EIYO. YU received research grants from Abbott Medical Japan and Medtronic, and lecture fees from NIPRO. HM is an endowed chair funded by TERUMO, Asahi Intecc, NIPRO, and Shimadzu Corporation, and received personal fees from Medtronic Japan, Japan Lifeline, and Abbott Medical Japan. YasushiS received grants from Abbott Medical Japan and Biotronik. The remaining authors declare that the research was conducted in the absence of any commercial or financial relationships that could be construed as a potential conflict of interest.

## Publisher's Note

All claims expressed in this article are solely those of the authors and do not necessarily represent those of their affiliated organizations, or those of the publisher, the editors and the reviewers. Any product that may be evaluated in this article, or claim that may be made by its manufacturer, is not guaranteed or endorsed by the publisher.
