# Supplementary material for: Pre-infarction Angina: Time Interval to Onset of Myocardial Infarction and Comorbidity Predictors
Source: Front Cardiovasc Med. 2022 May 26;9:867723. doi: 10.3389/fcvm.2022.867723 (PMC9204312; doi:10.3389/fcvm.2022.867723)
Supplement: Supplementary file 1 [file Table_1.DOCX]

**Supplemental Table 1.**

**Baseline characteristics of patients included and excluded from the analysis**

|  | **Patients analyzed** | **Patients excluded (Missing data in pre-infarction angina)** | **P value** | **Data missing (%)** |
| --- | --- | --- | --- | --- |
| Patients, n | 11117 | 976 |  |  |
| Age, y | 66.00 [58.00,75.00] | 72.00 [63.00,80.00] | <0.001 | 0 |
| Male sex | 8442 (75.9) | 654 (67.0) | <0.001 | 0 |
| Body mass index | 23.46 [21.44,25.71] | 22.98 [20.45,25.31] | <0.001 | 6.4 |
| Diabetes mellitus | 3655 (33.8) | 354 (40.5) | <0.001 | 3.3 |
| Hypertension | 6557 (60.8) | 585 (68.0) | <0.001 | 3.7 |
| Dyslipidemia | 4702 (44.2) | 315 (37.4) | <0.001 | 5.1 |
| Smoking | 6925 (63.9) | 447 (51.9) | <0.001 | 3.3 |
| Chronic kidney disease | 788 (7.3) | 118 (13.2) | <0.001 | 3.6 |
| Prior myocardial infarction | 1265 (11.7) | 135 (15.1) | 0.003 | 2.8 |
| Family history of MI | 967 (12.6) | 43 (10.5) | 0.239 | 33.3 |
| Cerebrovascular disease | 987 (9.2) | 153 (17.2) | <0.001 | 3.6 |
| Cancer | 622 (5.8) | 75 (8.4) | 0.002 | 3.6 |
| ASO | 274 (2.5) | 30 (3.4) | 0.171 | 3.6 |
| Hemoglobin, g/dL | 13.90 [12.40,15.20] | 12.60 [11.00,14.30] | <0.001 | 52 |
| Creatinine, mg/dL | 0.90 [0.70,1.10] | 1.00 [0.79,1.45] | <0.001 | 30.4 |
| Low density lipoprotein cholesterol, mg/dL | 121.00 [97.00,146.00] | 103.00 [81.75,131.70] | <0.001 | 63.9 |
| HbA1c, % | 5.60 [5.20,6.50] | 5.60 [5.20,6.70] | 0.214 | 27.2 |
| Culprit vessel |  |  |  |  |
| Right coronary artery | 3620 (35.6) | 284 (33.8) | 0.310 | 8.9 |
| Left anterior descending artery | 4863 (47.8) | 392 (46.6) | 0.533 | 8.9 |
| Left circumflex artery | 1608 (15.8) | 126 (15.0) | 0.562 | 8.9 |
| Left main trunk | 267 (2.6) | 49 (5.8) | <0.001 | 8.9 |
| Peak CK, IU/L | 1952.00 [908.25,3795.00] | 1786.00 [845.00,4272.00] | 0.977 | 6 |
| Peak CK-MB, IU/L | 172.60 [80.00,337.00] | 173.85 [74.60,365.00] | 0.407 | 14 |

Data are expressed as median [interquartile range] or number (percentage). Abbreviations: ASO, arteriosclerosis obliterans; CK, creatine kinase; CK-MB, creatine kinase myocardial band; MI, myocardial infarction.
